# Exosomes derived from Panax notoginseng promote osteogenic differentiation of rBMSCs via the PI3K/AKT signaling pathway

**DOI:** 10.3389/fphar.2025.1682548

**Published:** 2025-11-19

**Authors:** Nan Wu, Lintao Zhang, Hua Guo

**Affiliations:** 1 Department of Orthopedics, Xi’an Fifth Hospital, Xi’an, China; 2 School of Medicine, Xi’an Jiaotong University, Xi’an, China

**Keywords:** Panax notoginseng exosomes, osteoporosis, BMSCs, PI3K/Akt pathway, osteogenic differentiation, bone metabolism

## Abstract

**Objective:**

This study aims to investigate the effect of exosomes derived from Panax notoginseng on the osteogenic differentiation of rat bone marrow-derived mesenchymal stem cells (rBMSCs) and to elucidate the underlying intracellular signaling mechanisms.

**Methods:**

Exosomes from Panax notoginseng were isolated using differential centrifugation combined with sucrose density gradient centrifugation. The morphology of the exosomes was characterized by transmission electron microscopy (TEM), while size distribution and concentration were determined via nanoparticle tracking analysis (NTA). rBMSCs were isolated and identified by flow cytometry, and the uptake of fluorescently labeled Panax notoginseng exosomes by rBMSCs was confirmed using confocal microscopy. The optimal concentration of exosomes was determined using the CCK-8 assay. Osteogenic differentiation was evaluated by measuring alkaline phosphatase (ALP) activity, performing ALP staining, and conducting Alizarin Red S staining. The expression levels of osteogenic markers (collagen type I(COL1), ALP, osteopontin (OPN), and Runt-related transcription factor 2 (RUNX2)) were quantified at the mRNA (RT-qPCR) and protein (Westem blotting)levels. High-throughput RNA sequencing and bioinformatics analyses (Gene Ontology (GO),Kyoto Encyclopedia of Genes and Genomes (KEGG)) were employed to identify differentially expressed genes and enriched pathways. Key pathways were validated using specific inhibitors.

**Results:**

Exosomes derived from Panax notoginseng promote the osteogenic differentiation of rBMSCs through the activation of the PI3K/AKT signaling pathway. This study provides experimental evidence and theoretical support for the application of herbal exosomes in bone tissue engineering and the treatment of osteoporosis.

**Conclusion:**

Panax notoginseng exosomes promote osteogenic differentiation of rBMSCs by activating the PI3K/AKT pathway, providing experimental evidence and theoretical support for the application of herbal exosomes in bone tissue engineering and osteoporosis treatment.

## 1 Introduction

Osteoporosis (OP) is a common systemic metabolic bone disease characterized primarily by decreased bone mass, disruption of trabecular bone microarchitecture, and increased bone fragility, which seriously affects the physical and mental health as well as quality of life of middle-aged and elderly populations (Tarant et al., 2022; Liu et al., 2022). As a global public health concern, it is estimated that approximately 500 million people worldwide are affected by OP, with a significantly higher incidence rate in women than in men (Morin et al., 2025). Furthermore, an epidemiological study conducted in China in 2021 revealed that among adults aged 40 years and above, the prevalence of OP was 5.0% in men and as high as 20.6% in women (Wang et al., 2021).

Although currently available anti-OP medications in clinical practice can effectively delay bone loss and reduce fracture incidence, their efficacy and safety remain subject to certain limitations. Among these, bisphosphonates, as the most commonly used first-line therapeutic agents, primarily exert their effects by inhibiting bone resorption. However, long-term use may induce gastrointestinal adverse reactions, reduce patient compliance, and is significantly associated with serious complications such as atypical femoral fractures and medication-related osteonecrosis of the jaw (Aibar-Almazán et al., 2022; Alnajmi et al., 2024). Therefore, exploring novel therapeutic strategies with enhanced efficacy, improved safety profiles, and better compliance has become a research priority in the field of OP prevention and treatment.

In this context, therapeutic approaches based on bone marrow mesenchymal stem cells (BMSCs) have garnered increasing attention. BMSCs are pivotal cells in maintaining bone tissue homeostasis and, under normal circumstances, can differentiate into osteoblasts, thereby promoting bone formation and sustaining metabolic balance (Wei et al., 2024; Deng et al., 2025). However, in the pathological microenvironment of OP, the proliferation, survival, and osteogenic differentiation capacity of BMSCs are markedly impaired, resulting in insufficient bone formation and exacerbating metabolic dysregulation (Li et al., 2024). Consequently, effective modulation of the osteogenic differentiation of BMSCs has emerged as a critical entry point for ameliorating OP. In recent years, multiple studies have demonstrated that enhancing the relevant functions of BMSCs can significantly promote bone mass recovery (Wang et al., 2024b; Hu et al., 2024).

Meanwhile, the role of botanical drugs in the prevention and treatment of OP has garnered increasing attention. Their multi-target profiles and low toxicity offer new perspectives for correcting bone metabolic imbalance (Wan et al., 2024). Panax notoginseng, a widely used botanical drug in clinical practice, is traditionally recognized for “promoting blood circulation, resolving stasis, reducing swelling, and alleviating pain,” and it has been broadly applied to traumatic disorders (Wang et al., 2016). Modern pharmacological studies further substantiate that the principal active metabolites of Panax notoginseng, namely notoginsenosides, exhibit pronounced anti-inflammatory, antioxidant, and bone-repair–promoting activities (Li et al., 2025). Chemically, notoginsenosides are triterpenoid saponins composed of triterpenoid aglycones glycosidically linked to sugar moieties, forming amphipathic surface-active molecules. This architecture facilitates interactions with biological membranes and influences absorption, distribution, and metabolic processes, thereby modulating their pharmacological effects on bone metabolism (Zheng et al., 2024; Ko, 2024). Evidence indicates that notoginsenosides upregulate osteogenesis-related protein expression, restore bone metabolic homeostasis, inhibit bone loss, and promote bone repair (Huang and Li, 2020; Wang et al., 2022). In addition, their metabolites can markedly enhance the osteogenic differentiation of BMSCs, providing a solid theoretical basis for their application in OP prevention and treatment (Lee et al., 2023).

In recent years, extracellular vesicles (EVs), a class of naturally secreted nanoparticles, have attracted widespread attention in biological research. EVs primarily include exosomes, microvesicles, and apoptotic bodies. Among them, exosomes, with diameters ranging from approximately 30–150 nm, are released into the extracellular space through the fusion of multivesicular bodies with the plasma membrane followed by exocytosis (Doyle and Wang, 2019). Structurally, exosomes possess a characteristic lipid bilayer membrane that encapsulates various bioactive molecules, including proteins, lipids, mRNAs, miRNAs, and other non-coding RNAs. This unique architecture enables the transfer of genetic information and signaling molecules between cells, rendering exosomes key mediators of intercellular communication involved in numerous physiological and pathological processes, such as immune regulation, cell proliferation, differentiation, and apoptosis (Nicolini et al., 2021). In particular, within the bone microenvironment, exosomes derived from specific cell types play crucial roles in maintaining bone homeostasis and promoting bone repair by coordinating the balance between osteogenesis and osteoclastogenesis, stimulating angiogenesis, and modulating local immune responses (Liu et al., 2023). Despite the tremendous potential of animal-derived exosomes in regulating cellular functions and disease progression, their clinical application remains constrained by challenges such as complex isolation procedures, low yield, and potential immunogenicity (Zhang et al., 2021).

Therefore, researchers have turned their attention to plant-derived exosomes, which offer advantages such as low immunogenicity, high biocompatibility, and ease of isolation (Alzahrani et al., 2023). These properties have established them as an emerging strategy in the treatment of bone diseases and the development of botanical drugs (Jin et al., 2024; Mao et al., 2024). As natural intercellular transporters, plant exosomes can regulate the biological behavior of target cells by delivering nucleic acids, proteins, and chemical metabolites (Wang et al., 2024a). Several recent studies have preliminarily revealed the biological activities of Panax notoginseng-derived exosomes, including anti-inflammatory and anti-tumor effects (Li et al., 2023; Chen et al., 2024); however, whether they can be applied in the treatment of OP and the underlying mechanisms remain unclear. Given the crucial role of the PI3K/AKT signaling pathway in the osteogenic differentiation of BMSCs (Zou et al., 2025), we hypothesize that Panax notoginseng exosomes may promote osteogenesis by modulating this pathway.

Based on the above, the present study aims to systematically evaluate the regulatory effects of Panax notoginseng-derived EVs on the osteogenic differentiation of rBMSCs and to explore the potential molecular mechanisms, with a particular focus on the PI3K/AKT signaling pathway. This work is expected to provide experimental evidence and theoretical support for developing plant-derived EV–based novel therapeutic strategies for osteoporosis and to open new avenues for precision treatment of bone metabolic disorders.

## 2 Methods

### 2.1 Extraction and characterization of Panax notoginseng exosomes

This study used fresh Panax notoginseng (Burkill) F.H.Chen [Araliaceae] samples from Wenshan, Yunnan Province, China. The samples were carefully cleaned to remove surface impurities and dried under strictly controlled conditions to preserve their active metabolites. After drying, the rhizomes were cut into small segments and immediately immersed in pre-cooled sterile PBS buffer to prevent degradation of sensitive metabolites. The samples were then homogenized for 10 min, and the resulting slurry was filtered to remove coarse particles. To isolate exosomes and remove cellular debris, differential centrifugation combined with sucrose density gradient centrifugation was performed. The slurry was first centrifuged at 2,000 × g for 20 min, then at 10,000 × g for 60 min. The supernatant was subjected to sucrose density gradient centrifugation, where sucrose solutions (68% and 27% w/v) were layered in a tube, and the supernatant was added on top. After ultracentrifugation at 100,000 × g for 1.5 h, the fraction above the 68% sucrose layer was collected as crude exosomes. Further purification was done using a continuous sucrose gradient (8%, 30%, 45%, and 60% w/v), followed by ultracentrifugation at 200,000 × g for 1.5 h. The fraction between the 30% and 45% sucrose layers was collected as purified exosomes. All procedures were conducted at 4 °C to minimize degradation, and the purified exosomes were either used immediately or stored at −80 °C for long-term preservation, ensuring their integrity and stability.

Regarding extraction efficiency, approximately 1,000 g of fresh Panax notoginseng samples were used as raw material, yielding about 4 mL of purified Panax notoginseng exosome suspension with a concentration of approximately 7.3 × 10^11^ particles/mL, as determined by nanoparticle tracking analysis.

A 10 μL volume of purified Panax notoginseng exosome suspension was carefully dispensed onto a sealing film, and a copper grid was gently placed onto the droplet, allowing 20 min of incubation at ambient temperature to promote natural adsorption. Residual liquid was absorbed with filter paper, followed by air-drying of the copper grid at room temperature. Subsequently, the sample was fixed by applying 10 μL of 2% paraformaldehyde for 20 min, followed by negative staining with 10 μL of 2% phosphotungstic acid for 90 s. Excess staining solution was removed, and the copper grid was dried in the dark at room temperature. The prepared samples were analyzed for morphology and structure using a transmission electron microscope (HITACHI, Japan) at an accelerating voltage of 80–120 kV.

To evaluate the particle size and concentration of Panax notoginseng exosomes, a nanoparticle tracking analysis system (ParticleMetrix, Germany) was utilized. Exosome samples from Panax notoginseng were purified, passed through a 0.22 µm membrane filter, and diluted to a suitable concentration using PBS. Analyses were performed at ambient temperature with the nanoparticle tracking analysis system (ParticleMetrix, Germany). For each sample, a minimum of five fields of view were captured (60-s videos per field), and the mean particle size and concentration were determined using ZetaView software (version 8.05.10). All experiments were conducted in triplicate.

### 2.2 Culture and identification of rBMSCs

This study was approved by the Biomedical Ethics Committee of the Health Science Center, Xi’an Jiaotong University (approval number: XJTUAE20-3121). All experimental procedures strictly adhered to the institutional guidelines for animal ethics and welfare, and complied with the 3R principles of animal experimentation. Prior to the experiments, all 4-week-old SPF-grade Sprague-Dawley rats were deeply anesthetized via inhalation of 3.5% isoflurane to ensure complete analgesia throughout the procedure. Subsequently, the rats were euthanized by cervical dislocation in accordance with animal ethics requirements. Following euthanasia, the rat carcasses were immersed in 75% ethanol for 5 min to achieve surface disinfection. Under aseptic conditions, the soft tissues surrounding the femurs of the hind limbs were carefully dissected to isolate the femurs intact, which were then rinsed in sterile PBS. After trimming the epiphyses to expose the medullary cavity, the bone marrow was repeatedly flushed with complete culture medium containing 10% fetal bovine serum until the effluent became clear. The cell suspension was collected and centrifuged at 1,000 rpm for 5 min. The supernatant was discarded, and the cell pellet was resuspended in complete culture medium and seeded into culture dishes for cultivation at 37 °C under 5% CO_2_. The culture medium was replaced after 6 h of incubation to remove non-adherent cells, followed by medium changes every 2–3 days thereafter. Cells were passaged when reaching 80%–90% confluence. All experiments utilized cells from passages 3-5 during the logarithmic growth phase. To evaluate stem cell characteristics, cell surface markers including CD34, CD45, CD44, CD73, and CD105 were analyzed using flow cytometry (Cytek Aurora, United States).

### 2.3 Uptake of Panax notoginseng exosomes by rBMSCs

Panax notoginseng exosomes, isolated as previously described, were labeled with PKH67 fluorescent dye (Milian Bio, China) for 10 min at room temperature in light-protected conditions. The labeling reaction was quenched with 1% BSA. Subsequently, the exosomes were purified via ultracentrifugation (100,000 × g, 70 min, 4 °C) and rinsed with PBS to eliminate unbound dye. rBMSCs were seeded in culture dishes and maintained at 37 °C in a 5% CO_2_ atmosphere. These cells were incubated with Panax notoginseng exosomes at a concentration of 1 × 10^11^ particles/mL for 8 h. Post-incubation, the cells were washed three times with PBS, fixed in 4% paraformaldehyde for 15 min, and rinsed again with PBS three times after removing the fixative. The cells were then permeabilized using 0.2% Triton X-100 for 10 min, followed by three PBS washes. Cytoskeletal structures were visualized with phalloidin (Milian Bio, China), and nuclei were stained with DAPI (Solarbio, China). The samples were examined using a laser scanning confocal microscope (PerkinElmer, United States).

### 2.4 CCK-8 assay

The proliferation of rBMSCs in response to Panax notoginseng exosomes was evaluated via the CCK-8 assay. Cells were enzymatically dissociated, resuspended, and plated in 96-well plates at a density of 2 × 10^3^ cells per well. Following cell attachment, the culture medium was removed, and suspensions of Panax notoginseng exosomes at concentrations of 0, 1 × 10^6^, 1 × 10^7^, 1 × 10^8^, and 1 × 10^9^ particles/mL were introduced, with six replicate wells per condition. On days 1, 3, 5, and 7 of incubation, the well contents were aspirated, and 100 μL of serum-free medium containing 10 μL of CCK-8 reagent was added to each well. After a 2-h incubation in the absence of light, absorbance at 450 nm was quantified using a microplate reader.

### 2.5 ALP activity assay and staining analysis

To evaluate the influence of Panax notoginseng exosomes on early osteogenic differentiation, three groups were established: a blank control group (NC), devoid of Panax notoginseng exosomes and osteogenic induction medium; a control group (CON), supplemented solely with osteogenic induction medium; and an exosome group (EXO), incorporating both Panax notoginseng exosomes and osteogenic induction medium. On days 7 and 14 of culture, alkaline phosphatase (ALP) activity and staining assays were conducted. For the ALP activity assay, cells were lysed using 80 μL of lysis buffer (Solarbio, China) for 15 min, then centrifuged at 12,000 × g at 4 °C for 15 min to obtain the supernatant. Protein concentrations were quantified with a BCA protein assay kit (Beyotime, China), and samples were diluted five-fold in lysis buffer prior to measuring ALP activity with an alkaline phosphatase assay kit (Beyotime, China). For ALP staining, cells were rinsed with PBS, fixed in 1 mL of 4% paraformaldehyde at room temperature for 30 min, and stained using a BCIP/NBT alkaline phosphatase staining kit (Beyotime, China). Stained samples were visualized and photographed under an inverted microscope (Olympus Corporation, Japan).

### 2.6 Alizarin Red S staining and quantitative analysis

To assess the role of Panax notoginseng exosomes in late osteogenic differentiation, three experimental groups were established: a blank control group (NC), lacking both Panax notoginseng exosomes and osteogenic induction medium; a control group (CON), supplemented exclusively with osteogenic induction medium; and an exosome group (EXO), containing both Panax notoginseng exosomes and osteogenic induction medium. Alizarin Red S staining and quantitative analysis of mineralized matrix deposition were conducted after 14 and 28 days of culture. For the staining procedure, cells were rinsed three times with PBS, fixed in 1 mL of 4% paraformaldehyde at room temperature for 20 min, and then rinsed again with PBS. Subsequently, 1 mL of Alizarin Red S staining solution (Solarbio, China) was added to each well, and cells were incubated in the dark at room temperature for 30 min. The staining solution was then removed, and cells were washed twice with sterile water. Staining outcomes were visualized and photographed using an inverted phase-contrast microscope (Olympus Corporation, Japan). For quantitative evaluation of mineralized matrix, 1 mL of 10% cetylpyridinium chloride solution (CPC, Solarbio, China) was added to each well, followed by oscillation for 30 min to fully dissolve the stain. Absorbance was measured at 562 nm using a microplate reader. In this study, Alizarin Red S staining and CPC absorbance measurements were used solely for relative comparisons between groups and were not interpreted as absolute quantitative data.

### 2.7 RT-qPCR

The study was organized into two groups: a control group (CON), provided solely with osteogenic induction medium, and an exosome-treated group (EXO), supplemented with both osteogenic induction medium and Panax notoginseng exosomes. Following 14 days of culture, total RNA was isolated using Trizol reagent (ABI, United States). cDNA was generated with an RNA reverse transcription kit (Takara, Japan). Quantitative mRNA analysis was conducted using SYBR Green/ROX RT-qPCR Mix (Takara, Japan) on a StepOnePlus™ Real-Time PCR System (Thermo Fisher Scientific, United States). The primer sequences used are as follows.

ALP: 5′TCA​TTC​CCA​CGT​TTT​CAC​ATT​C 3′和5′GTT​GTT​GTG​AGC​GTA​ATC​TAC​C 3';

COL1:5′TGAACGTGGTGTACAAGGTC3′和5′CCATCTTTACCAGGAGAACCAT3';

RUNX2: 5′AAG​GCA​CAG​ACA​GAA​GCT​TGA3′和5′AGG​ACT​TGG​TGC​AGA​GTT​CAG 3';

OPN: 5′ACA​CAT​ATG​ATG​GCC​GAG​GT3′和5′TCA​TCC​AGC​TGA​CTC​GTT​TC3';

GAPDH: 5′TGG​AAA​GCT​GTG​GCG​TGA​TG3′和5′TAC​TTG​GCA​GGT​TTC​TCC​AGG 3'.

Data analysis was performed using the 2^−ΔΔCt^ method. Each assay was conducted in triplicate, and the results were averaged.

### 2.8 Western blot

The experiment was divided into a control group (CON, containing only osteogenic induction medium) and an exosome-treated group (EXO, containing osteogenic induction medium and Panax notoginseng exosomes). After 14 days of cell culture, cells were lysed on ice for 20 min, and the supernatant was collected by centrifugation to prepare protein samples. Protein concentration was determined using a BCA kit (Beyotime, China). Equal amounts of protein samples were separated by 12% SDS-PAGE and transferred onto a PVDF membrane, followed by blocking with 5% non-fat milk at room temperature for 2 h. Target bands corresponding to the molecular weights of ALP, COL1, RUNX2, OPN, and GAPDH.

(Proteintech, United States) were excised and incubated with respective primary antibodies at 4 °C overnight. After washing with TBST (Beyotime, China) for 30 min, samples were incubated with secondary antibodies at room temperature for 1 h. Protein expression levels were visualized and analyzed using an ECL chemiluminescence kit (Yuheng Bio, China).

### 2.9 Transcriptomic sequencing and pathway analysis

Total RNA was isolated from rBMSCs exposed to Panax notoginseng exosomes or PBS using the Animal RNA Isolation Kit (Mjzol, China), with RNA quality evaluated through the Agilent 2,100 Bioanalyzer (Agilent, United States). mRNA libraries were then prepared in accordance with the TruSeq Stranded mRNA LT Sample Prep Kit (Illumina, United States) protocols and sequenced on the HiSeq™ 2,500 platform (Illumina, United States). Differential expression analysis was conducted across both treatment groups, applying a threshold of fold change >2 and q-value <0.05. Genes exhibiting significant differential expression were grouped based on similar expression profiles using clustering analysis and visualized via volcano plots and heatmaps in R. Additionally, Gene Ontology (GO) and Kyoto Encyclopedia of Genes and Genomes (KEGG) pathway enrichment analyses were performed to identify enriched terms potentially linked to Panax notoginseng exosome treatment, with findings displayed in enrichment bubble plots.

### 2.10 LY294002 treatments

LY294002, a specific inhibitor of the PI3K/AKT signaling pathway, was used in this study. Three experimental groups were established: CON group (PBS treatment), EXO group (Panax notoginseng exosome treatment), and EXO + LY group (Panax notoginseng exosome combined with LY294002 treatment). After 14 days of induction culture, Real-Time PCR and Western blot were employed to detect the expression levels of relevant genes and proteins.

## 3 Result

### 3.1 Extraction and characterization of panax notoginseng exosomes and rBMSCs

**FIGURE 1:**
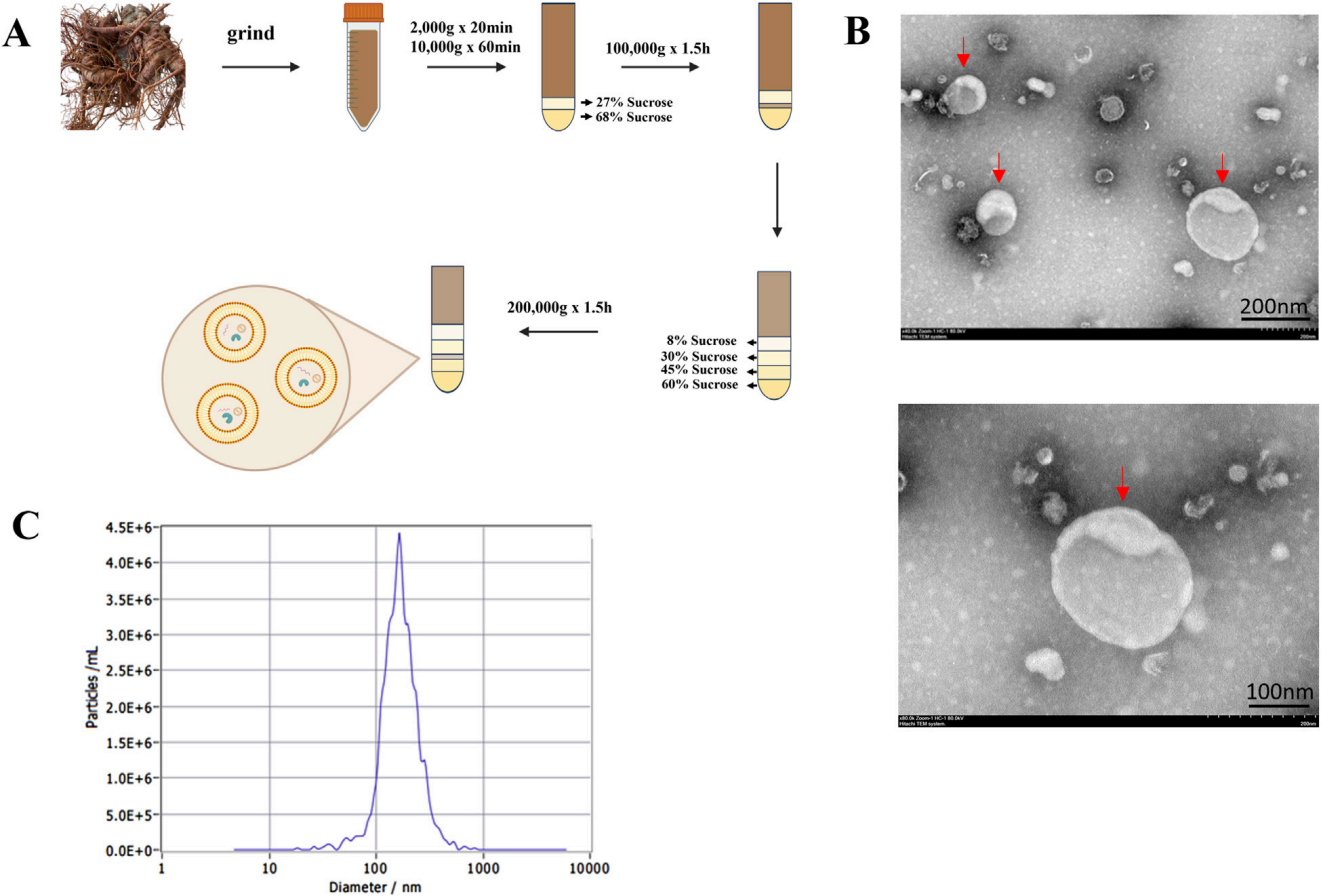
Extraction and characterization of Panax notoginseng exosomes. **(A)** Schematic diagram of the extraction process of Panax notoginseng exosomes. **(B)** TEM observations of Panax notoginseng exosomes. **(C)** NTA results of Panax notoginseng exosomes.

**FIGURE 2:**
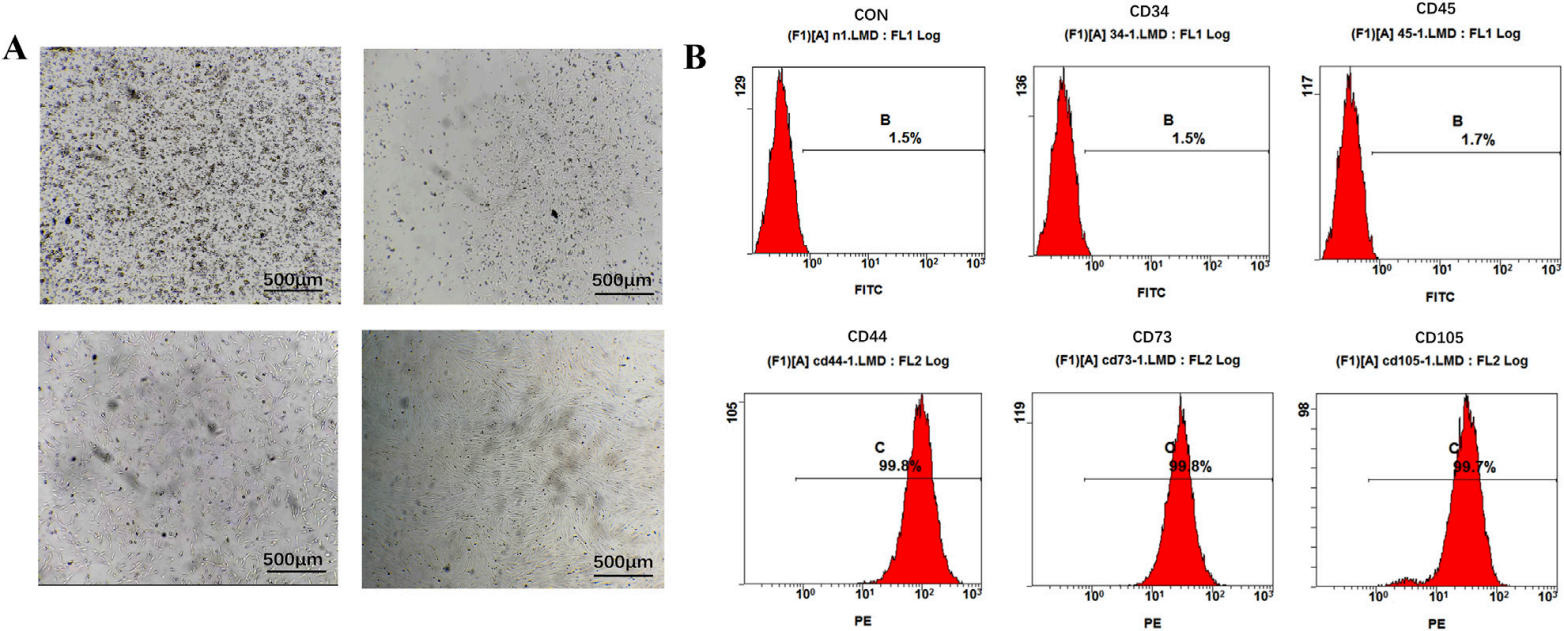
Extraction and characterization of rBMSCs. **(A)** Culture of rBMSCs. **(B)** Flow cytometry analysis of surface markers of rBMSCs.

In this study, Panax notoginseng exosomes were successfully extracted using differential centrifugation combined with sucrose density gradient centrifugation, with the detailed extraction procedure shown in Figure 1A. TEM results, as shown in Figure 1B, revealed that the extracted Panax notoginseng exosomes exhibited a typical saucer-like structure. NTA results, as shown in Figure 1C, indicated a uniform particle size distribution of approximately 162.5 nm and a concentration of 7.3 × 10^11^ particles/mL. rBMSCs were isolated and cultured, with microscopic results shown in Figure 2A: 6 hours post-seeding, few adherent cells were observed, displaying short spindle or round morphology; after 4 days of culture, cell numbers significantly increased, transitioning to a long spindle shape and forming colonies; by day seven, cells exhibited a uniform long spindle morphology, reaching 80%–90% confluence, suitable for passaging; P3 generation cells showed uniform morphology with clear boundaries. Flow cytometry analysis of rBMSC surface markers, as shown in Figure 2B, demonstrated high expression of CD44, CD73, and CD105 (positive rates of 99.8%, 99.8%, and 99.7%, respectively) and low expression of CD34 and CD45 (positive rates of 1.5% and 1.7%, respectively), consistent with the phenotypic identification standards for rBMSCs (Zhao et al., 2020). These results confirm the successful extraction of high-purity Panax notoginseng exosomes and standard-compliant rBMSCs, laying the foundation for subsequent studies on the biological functions and mechanisms of Panax notoginseng exosomes.

### 3.2 Uptake of Panax notoginseng exosomes by rBMSCs and their effects on cell activity

**FIGURE 3:**
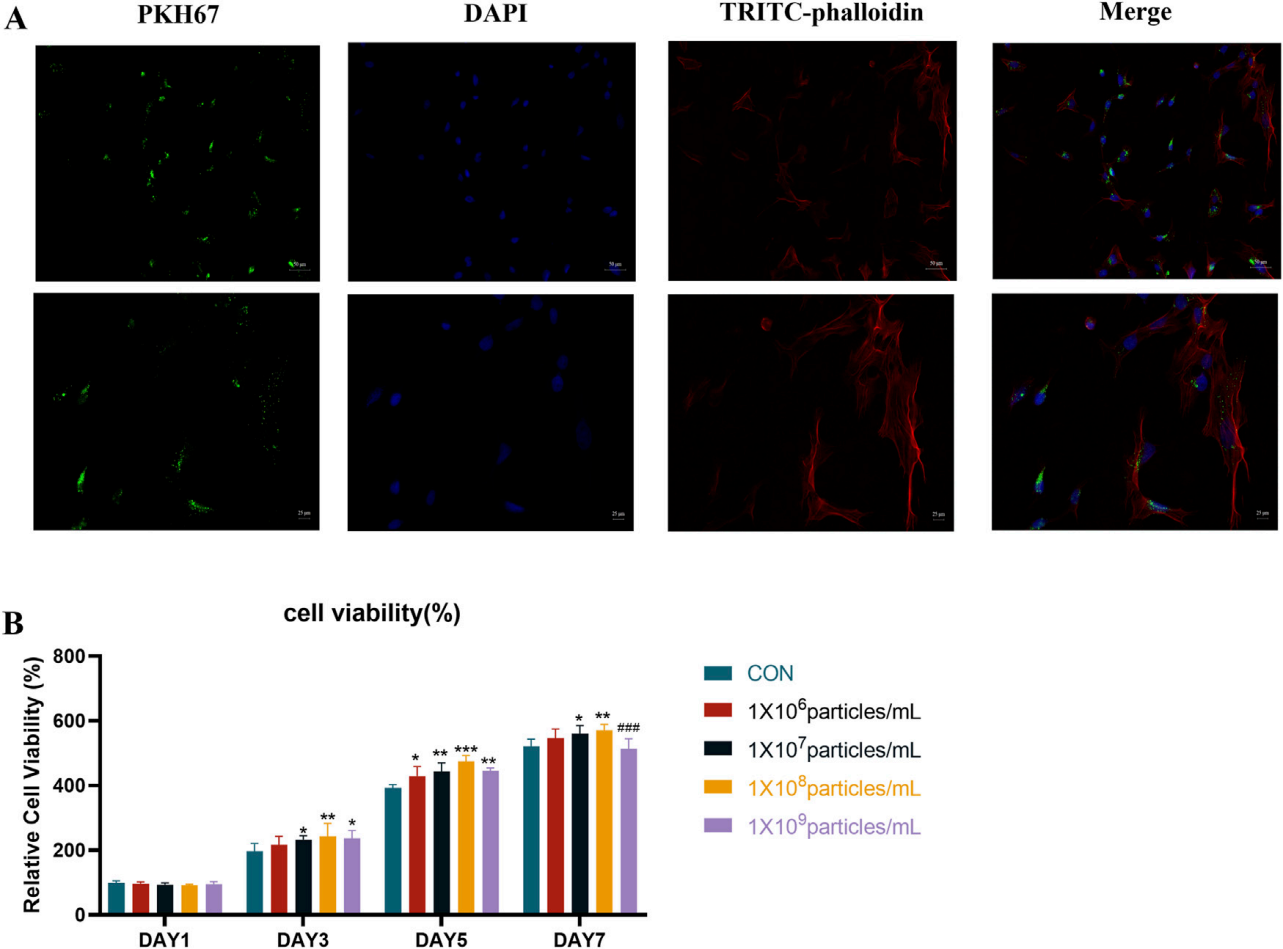
Uptake of Panax notoginseng-derived exosomes by rBMSCs. **(A)** Confocal microscopy fluorescence images of rBMSCs co-cultured with PKH67-labeled Panax notoginseng exosomes for 8 h. **(B)** Effect of Panax notoginseng exosomes on rBMSC viability assessed by CCK-8 assay (mean ± SD, n = 6). Statistical analysis: Two-way ANOVA followed by Tukey’s *post hoc* test. ###P < 0.001 vs. 1 × 10^8 particles/mL group; P < 0.05, *P < 0.01, **P < 0.001 vs. CON group.

To investigate whether Panax notoginseng exosomes are taken up by rBMSCs, this study labeled the lipid bilayer of Panax notoginseng exosomes with the lipophilic fluorescent dye PKH67 and subsequently co-cultured the labeled exosomes with rBMSCs. The cytoskeleton was stained with phalloidin, and the nucleus was stained with DAPI to determine the intracellular localization of Panax notoginseng exosomes. Confocal laser microscopy observations (Figure 3A) revealed blue fluorescence in the nucleus, red fluorescence in the cytoskeleton, and green fluorescence from Panax notoginseng exosomes, indicating that Panax notoginseng exosomes can be effectively taken up by rBMSCs and localized within the cytoplasm.

To examine the influence of Panax notoginseng exosomes on rBMSC proliferation, the CCK-8 assay was conducted to evaluate varying concentrations of exosomes (1 × 10^6^, 1 × 10^7^, 1 × 10^8^, 1 × 10^9^ particles/mL), with outcomes presented in Figure 3B. On day 1, cell viability showed no notable differences between the treatment groups and the CON group. Starting from day 3, a dose-dependent increase in proliferation was observed with Panax notoginseng exosomes, with the 1 × 10^8^ particles/mL group demonstrating the most pronounced effect. By day 5, this dose-dependent pattern continued, though the 1 × 10^9^ particles/mL group exhibited significantly reduced viability compared to the 1 × 10^8^ particles/mL group. On day 7, the 1 × 10^7^, 1 × 10^8^, and 1 × 10^9^ particles/mL groups displayed significantly enhanced viability relative to the CON group, whereas the 1 × 10^6^ particles/mL group showed no substantial change. Notably, viability in the 1 × 10^9^ particles/mL group remained markedly lower than in the 1 × 10^8^ particles/mL group. Following comprehensive evaluation, 1 × 10^8^ particles/mL was determined as the optimal concentration for subsequent experiments.

### 3.3 Panax notoginseng exosomes significantly enhance the osteogenic differentiation and mineralization capacity of rBMSCs

**FIGURE 4:**
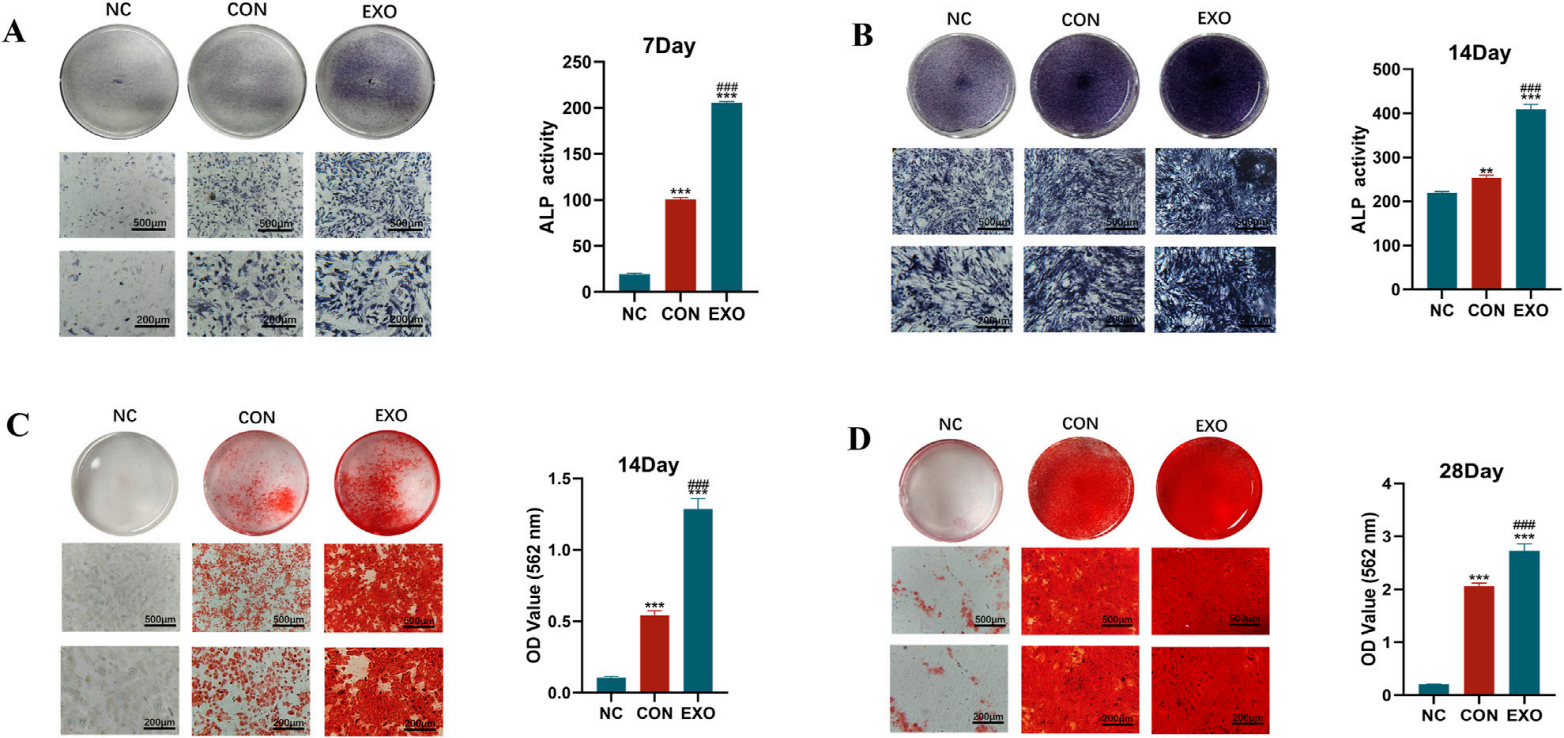
Effects of Panax notoginseng-derived exosomes on ALP activity and mineralized matrix deposition in rBMSCs. **(A)** ALP staining images and quantitative analysis of ALP activity in rBMSCs on day 7. **(B)** ALP staining images and quantitative analysis of ALP activity in rBMSCs on day 14. **(C)** Alizarin Red S staining images of mineralized nodules and quantitative analysis in rBMSCs on day 14. **(D)** Alizarin Red S staining images of mineralized nodules and quantitative analysis in rBMSCs on day 28. (mean ± SD, n = 3). Statistical analysis: One-way ANOVA followed by Tukey’s *post hoc* test. ###P < 0.001 vs. CON group; *P < 0.01, **P < 0.001 vs. NC group.

To investigate the influence of Panax notoginseng exosomes on the osteogenic differentiation and mineralized matrix formation of rBMSCs, an integrated analysis was performed. Results from ALP staining and quantitative assays (Figures 4A,B) revealed that on day 7, both ALP staining intensity and enzymatic activity in the EXO group were markedly elevated compared to the NC and CON groups. By day 14, ALP expression in the EXO group continued to exhibit significantly higher levels relative to the NC and CON groups.

Alizarin Red S staining, paired with CPC dissolution-based quantitative assessment (Figures 4C,D), substantiated the presence of mineralized nodules. On day 14, the NC group displayed no discernible red nodules, whereas all other groups exhibited prominent red calcium nodules. The CON group showed greater staining intensity and a higher count of calcium nodules compared to the NC group, yet these metrics remained inferior to those observed in the EXO group. Quantitative analysis using CPC further validated that calcium deposition in the EXO group consistently surpassed that in the NC and CON groups across all evaluated time points.

### 3.4 Panax notoginseng exosomes enhance the expression of osteogenic markers at the transcriptional and translational levels

**FIGURE 5:**
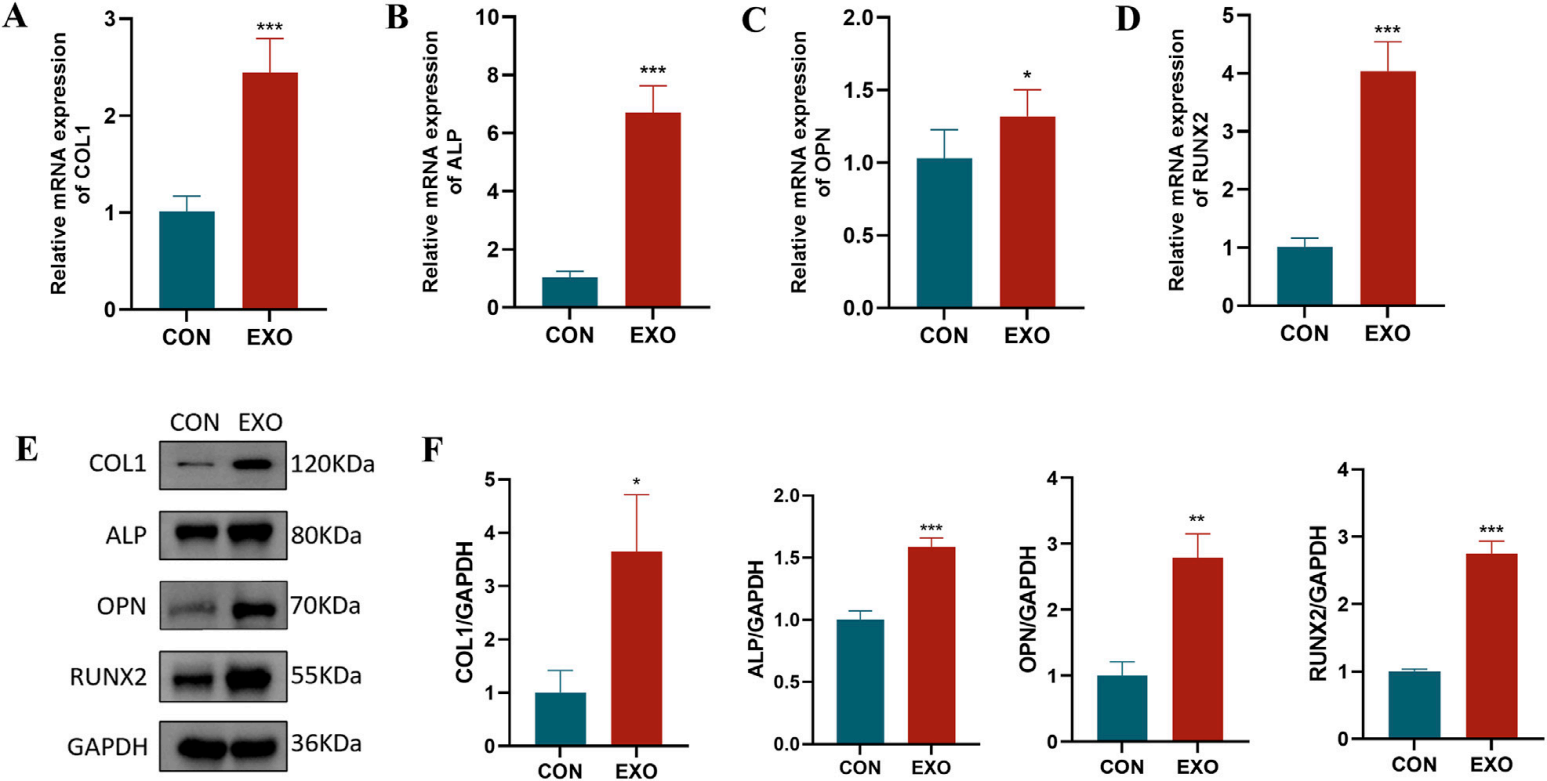
Effects of Panax notoginseng-derived exosomes on osteogenic protein and gene expression in rBMSCs. RT-qPCR was used to detect mRNA expression changes of osteogenic genes COL1 **(A)**, ALP **(B)**, OPN **(C)**, and RUNX2 **(D)** in rBMSCs following treatment with Panax notoginseng exosomes. **(E)** Western blot analysis of osteogenic protein (COL1, ALP, OPN, RUNX2) expression levels in rBMSCs treated with PBS or Panax notoginseng exosomes, with grayscale quantitative analysis of protein bands for COL1, ALP, OPN, and RUNX2 **(F)**. (mean ± SD, n = 3). Statistical analysis: Independent samples t-test. P < 0.05, *P < 0.01, **P < 0.001 vs. CON group.

To examine the impact of Panax notoginseng exosomes on osteogenesis-related gene and protein expression in rBMSCs, cells were cultured in the CON and EXO groups. RT-qPCR assays (Figures 5A–D) indicated that mRNA levels of key osteogenic genes, including COL1, ALP, OPN, and RUNX2, were markedly elevated in the EXO group relative to the CON group. Western blot analysis (Figures 5E,F) corroborated these findings, revealing increased protein expression of COL1, ALP, OPN, and RUNX2 in the EXO group, aligning with the RT-qPCR data. These results demonstrate that Panax notoginseng exosomes effectively enhance the expression of osteogenesis-related factors at both transcriptional and translational levels.

### 3.5 Transcriptomic analysis revealed pathways potentially regulated by panax notoginseng exosomes

**FIGURE 6:**
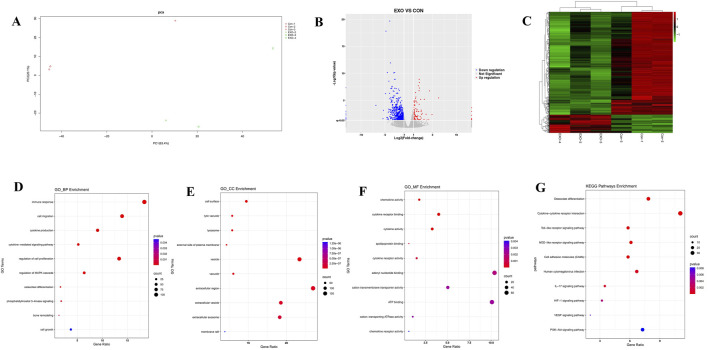
Transcriptomic profiling of rBMSCs comparing the CON and EXO groups. **(A)** PCA plot illustrates clear separation of cell clusters between the CON and EXO groups. **(B)** Volcano plot highlights differentially expressed genes (DEGs), with red dots indicating upregulated genes and blue dots denoting downregulated genes (threshold: |Log2 fold change| > 1, p < 0.05). **(C)** Heatmap presents hierarchical clustering of DEGs across the CON and EXO groups, elucidating global expression differences. GO enrichment analysis of upregulated genes identifies significantly enriched terms within biological process **(D)**, cellular component **(E)**, and molecular function categories **(F)**. **(G)** KEGG pathway analysis reveals that Panax notoginseng exosome treatment markedly activates critical pathways, notably the PI3K-Akt pathway (p < 0.05).

To elucidate the molecular mechanisms governing the osteogenic differentiation of rBMSCs induced by Panax notoginseng exosomes, principal component analysis (PCA) was conducted on transcriptomic sequencing data to evaluate sample variability. The PCA outcomes (Figure 6A) revealed distinct separation between the CON and EXO groups, with strong consistency within each group. Differentially expressed genes (DEGs) were identified using DESeq2 with rigorous criteria (|log2 fold change| > 1, q-value <0.05), yielding 2,298 DEGs, of which 1,547 were upregulated and 751 were downregulated. Volcano plots (Figure 6B) and heatmaps (Figure 6C) effectively visualized the distribution and expression profiles of these DEGs. GO enrichment analysis (Figures 6D–F) indicated that DEGs in biological processes (BP) were predominantly enriched in bone metabolism, immune and inflammatory responses, cell migration, and proliferation; in cellular components (CC), enrichment was observed in the extracellular matrix, membrane-associated regions, and intracellular vesicles; and in molecular functions (MF), DEGs were notably enriched in ion transport, cytokine activity, and lipid binding. Furthermore, KEGG pathway analysis (Figure 6G) highlighted significant activation of the PI3K/AKT pathway.

### 3.6 Panax notoginseng exosomes may promote the osteogenic differentiation of BMSCs by activating the PI3K/AKT pathway

**FIGURE 7:**
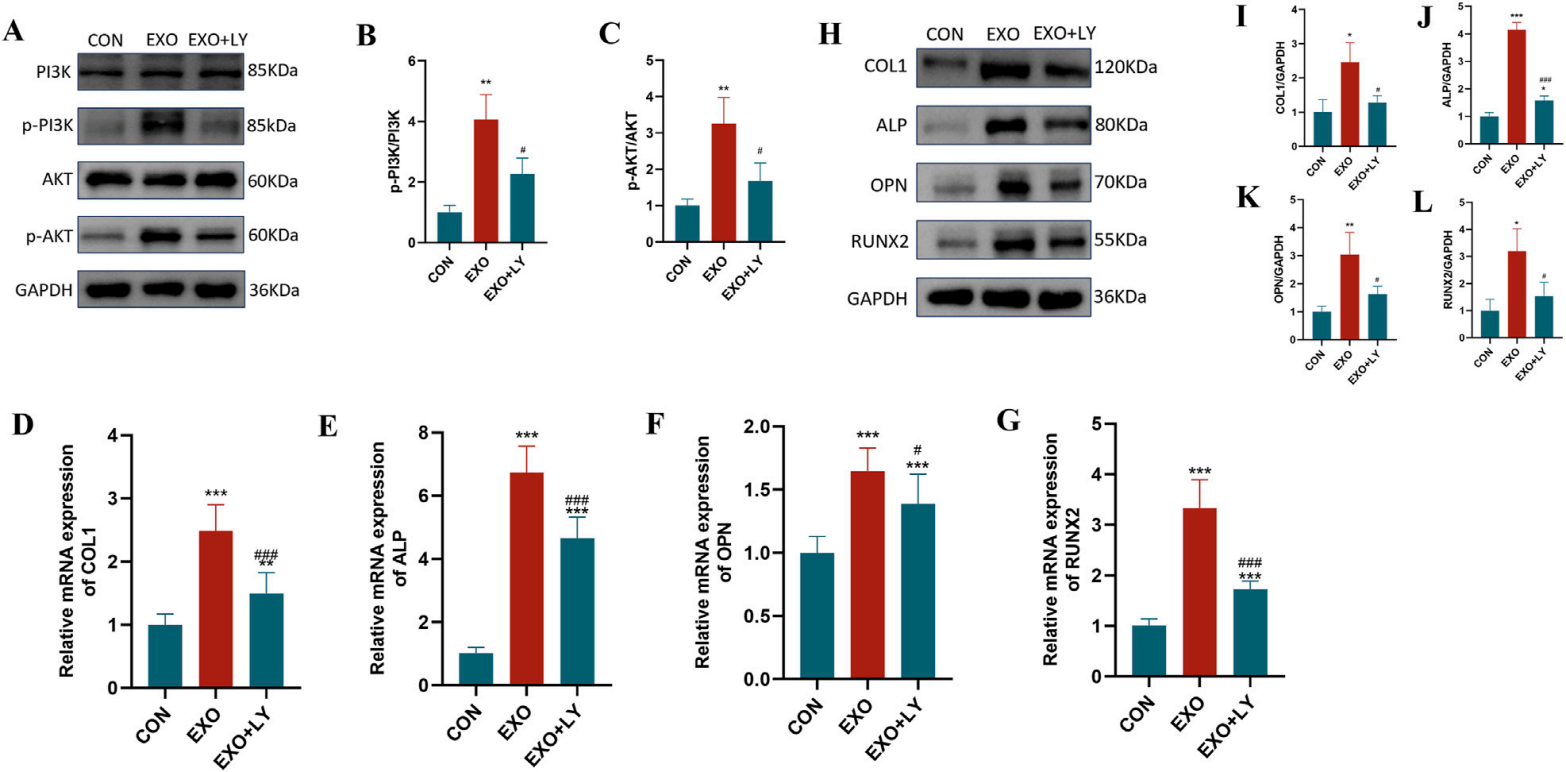
Effects of LY294002 on Panax notoginseng exosome-mediated osteogenic differentiation of rBMSCs. **(A)** Western blot analysis of phosphorylated PI3K (p-PI3K), total PI3K, phosphorylated Akt (p-Akt), and total Akt protein expression levels in rBMSCs from the control (CON), Panax notoginseng exosome (EXO), and Panax notoginseng exosome + PI3K/Akt inhibitor LY294002 (EXO + LY) groups. **(B,C)** Grayscale quantitative analysis of p-PI3K/PI3K and p-Akt/Akt ratios. **(D–G)** qRT-PCR detection of mRNA expression levels of osteogenic genes COL1 **(D)**, ALP **(E)**, OPN **(F)**, and RUNX2 **(G)** in rBMSCs from the CON, EXO, and EXO + LY groups. **(H)** Western blot analysis of osteogenic protein (COL1, ALP, OPN, RUNX2) expression levels in rBMSCs from the CON, EXO, and EXO + LY groups, with grayscale quantitative analysis of protein bands for COL1 **(I)**, ALP **(J)**, OPN **(K)**, and RUNX2 **(L)**. (mean ± SD, n = 3). Statistical analysis was performed using one-way ANOVA followed by Tukey’s *post hoc* test. P < 0.05, *P < 0.01, **P < 0.001 vs. CON group; #P < 0.05, ###P < 0.001 vs. EXO group.

To confirm the involvement of the PI3K/AKT pathway in Panax notoginseng exosome-mediated osteogenic differentiation of rBMSCs, experiments were performed using the PI3K/AKT-specific inhibitor LY294002. Western blot analysis (Figures 7A–C) revealed markedly elevated levels of phosphorylated PI3K (p-PI3K) and phosphorylated AKT (p-AKT) in the EXO group compared to the CON group, with LY294002 treatment (EXO + LY group) substantially diminishing this elevation. RT-qPCR assays (Figures 7D–G) demonstrated that mRNA expression of osteogenesis-related genes (COL1, ALP, OPN, RUNX2) was significantly higher in the EXO group than in the CON group, and this increase was notably reduced by LY294002 intervention. Furthermore, Western blot analysis of osteogenesis-related proteins (Figures 7H–L) indicated that protein levels of COL1, ALP, OPN, and RUNX2 were prominently increased in the EXO group, while LY294002 treatment (EXO + LY group) effectively suppressed their expression. These findings suggest that Panax notoginseng exosomes enhance osteogenesis-related gene and protein expression in rBMSCs, promoting osteogenic differentiation, likely via activation of the PI3K/AKT pathway.

## 4 Discussion

Panax notoginseng, as a traditional botanical drug, is widely used in the treatment of cardiovascular, hepatorenal, and orthopedic diseases, and its principal bioactive metabolites, saponins, have been demonstrated to exert significant biological effects (Wang et al., 2016; Guo et al., 2019; Han et al., 2022). Studies have shown that notoginsenoside R1 can exert anti-osteoporotic effects by promoting the osteogenic differentiation of MC3T3-E1 cells (Liu et al., 2016). In recent years, plant-derived exosomes, as novel bioactive carriers, have garnered widespread attention. Previous studies have demonstrated that Panax notoginseng-derived exosomes can inhibit the proliferation and migration of squamous cell carcinoma and alleviate ischemia-reperfusion injury through modulation of microglial cell phenotypes (Li et al., 2023; Chen et al., 2024). These findings suggest that Panax notoginseng-derived exosomes play an important biological role in the regulation of cellular functions and disease prevention. However, their specific roles and molecular mechanisms in the regulation of bone metabolism remain unclear. This study systematically investigated the effects of Panax notoginseng-derived exosomes on the osteogenic differentiation of rBMSCs and the underlying mechanisms. BMSCs possess multipotent differentiation capacity and can differentiate into osteoblasts, adipocytes, and chondrocytes, serving as key effector cells in the bone formation process (Gholami Farashah et al., 2023). This study provides the first evidence that Panax notoginseng-derived exosomes can significantly promote the osteogenic differentiation of rBMSCs through activation of the PI3K/AKT signaling pathway, offering a novel perspective for functional studies of plant-derived exosomes in bone metabolism regulation and establishing a theoretical foundation for the development of natural product-based bone-targeting therapeutic strategies.

Panax notoginseng exosomes extracted by differential centrifugation combined with sucrose gradient density centrifugation exhibited high purity, with an average particle size of approximately 162.5 nm and a concentration of 7.3 × 10^11^ particles/mL. TEM observation revealed a typical cup-shaped structure, consistent with the morphological characteristics of exosomes. To confirm whether Panax notoginseng exosomes could be effectively taken up by rBMSCs to exert potential biological functions, uptake experiments were conducted, and the results demonstrated that rBMSCs could efficiently internalize Panax notoginseng exosomes, providing a necessary prerequisite and foundation for subsequent functional studies. CCK-8 assays showed that within the concentration range of 1 × 10^7^ to 1 × 10^8^ particles/mL, Panax notoginseng exosomes significantly enhanced the proliferative activity of rBMSCs, with the optimal effect observed at 1 × 10^8^ particles/mL. However, at a concentration of 1 × 10^9^ particles/mL, cell viability decreased, suggesting that high concentrations of exosomes may induce cytotoxicity or metabolic burden. Therefore, optimizing the effective concentration of Panax notoginseng exosomes is critical for functional studies.

Osteogenic differentiation is a multifaceted, tightly regulated process involving multiple stages. This investigation assessed the influence of Panax notoginseng exosomes on the osteogenic differentiation of rBMSCs through techniques such as ALP staining, Alizarin Red S staining, RT-qPCR, and Western blot. ALP staining indicated that ALP activity in the EXO group was markedly elevated compared to the CON group on days 7 and 14, demonstrating that Panax notoginseng exosomes substantially enhance early osteogenic differentiation of rBMSCs. Elevated ALP activity, a recognized early marker of osteogenic differentiation, signifies the onset of this process (Hatta et al., 2002). Alizarin Red S staining further revealed significantly greater calcium nodule formation in the EXO group compared to the control group on days 14 and 28, indicating that Panax notoginseng exosomes promote late-stage osteogenic differentiation of rBMSCs. Calcium nodule formation, a critical hallmark of late-stage osteogenic differentiation, reflects extracellular matrix mineralization (Bernar et al., 2022). These findings collectively underscore the stimulatory role of Panax notoginseng exosomes across the osteogenic differentiation process. Furthermore, RT-qPCR and Western blot analyses demonstrated that mRNA and protein expression levels of ALP, COL1, RUNX2, and OPN were prominently upregulated in the EXO group, suggesting that Panax notoginseng exosomes enhance osteogenic differentiation of rBMSCs by modulating the expression of key osteogenesis-related genes and proteins.

Transcriptomic sequencing and bioinformatics analysis revealed pronounced gene expression differences between the EXO and CON groups, identifying 2,298 DEGs, with 1,547 upregulated and 751 downregulated. GO enrichment analysis demonstrated that these DEGs were predominantly enriched in metabolism-related processes, including bone remodeling, osteoclast differentiation, MAPK signaling cascade regulation, and the PI3K pathway, alongside biological processes such as inflammatory response, immune response, and cell proliferation regulation, which are intricately linked to osteogenic differentiation and microenvironment modulation. KEGG pathway analysis further highlighted significant enrichment in pathways such as osteoclast differentiation, cytokine-cytokine receptor interaction, Toll-like receptor signaling pathway, NOD-like receptor signaling pathway, cell adhesion molecules, human cytomegalovirus infection, IL-17 signaling pathway, HIF-1 signaling pathway, VEGF signaling pathway, and PI3K/AKT signaling pathway. Despite its lower ranking in KEGG enrichment, the PI3K/AKT pathway exhibited a strong association with GO terms related to bone remodeling and osteogenic differentiation, markedly enriching key osteogenic genes like ALP, COL1A1, RUNX2, and OPN. This pathway was prioritized for investigation due to robust experimental validation using the LY294002 inhibitor and supporting literature. Conversely, higher-ranked pathways, such as osteoclast differentiation and cytokine-cytokine receptor interaction, were more closely tied to broad inflammatory and immune processes, aligning with GO terms for inflammatory and immune responses but showing limited specificity for osteogenic differentiation, thus receiving lower priority. The co-enrichment of inflammation- and oxidative stress-related pathways (e.g., IL-17 and Toll-like receptor signaling pathways) in KEGG analysis suggested that Panax notoginseng exosomes may mitigate BMSC senescence and enhance the osteogenic microenvironment by attenuating inflammatory responses and oxidative stress. Experimental validation through Western blot and qRT-PCR confirmed that Panax notoginseng exosomes significantly enhanced phosphorylation of PI3K and AKT proteins and expression of osteogenic markers (e.g., COL1, ALP, RUNX2, OPN). However, these effects were substantially diminished by the PI3K/AKT-specific inhibitor LY294002, underscoring the critical role of the PI3K/AKT pathway in mediating Panax notoginseng exosome-driven osteogenic differentiation in rBMSCs.

This investigation has yielded notable advancements, yet several limitations persist Firstly, the pivotal role of the PI3K/AKT pathway in mediating the osteogenic effects of Panax notoginseng exosomes has been substantiated solely through *in vitro* cellular assays, necessitating further validation in animal models or clinical trials. Secondly, the precise bioactive ​metabolites​ within Panax notoginseng exosomes driving these effects remain largely unidentified, which could offer more specific targets for developing therapeutic strategies for OP. Furthermore, the regulatory interplay between the PI3K/AKT pathway and other metabolic pathways requires deeper exploration to fully clarify the molecular mechanisms governing Panax notoginseng exosome-mediated bone metabolism regulation.

## Data Availability

The data presented in the study are deposited in the Figshare repository, available at doi: 10.6084/m9.figshare.30605699.
